# Neurofibromatosis I and multiple sclerosis

**DOI:** 10.1186/s13023-020-01463-z

**Published:** 2020-07-14

**Authors:** Christina Bergqvist, François Hemery, Salah Ferkal, Pierre Wolkenstein

**Affiliations:** 1 Faculty of Medicine, Universite Paris-Est Creteil , Créteil, France; 2 Department of Dermatology, Hopital Henri Mondor, Assistance Publique-Hôpital Paris (AP-HP), Créteil, France; 3 Department of Medical Informatics, Hôpital Henri-Mondor, Assistance Publique-Hôpitaux Paris (AP-HP), Créteil, France; 4 INSERM, Centre d’Investigation Clinique 006, Hôpital Henri-Mondor, Assistance Publique-Hôpitaux Paris (AP-HP), Referral Center of Neurofibromatosis, Créteil, France

**Keywords:** Neurofibromatosis 1, Multiple sclerosis, Autoimmune disease, Genodermatosis

## Abstract

Neurofibromatosis 1 (NF1) is one of the most common autosomal dominant genetic disorders with a birth incidence as high as 1:2000. It is caused by mutations in the *NF1* gene on chromosome 17 which encodes neurofibromin, a regulator of neuronal differentiation. While NF1 individuals are predisposed to develop benign and malignant nervous system tumors, various non-tumoral neurological conditions including multiple sclerosis (MS) have also been reported to occur more frequently in NF1. The number of epidemiologic studies on MS in NF1 individuals is very limited. The aim of this study was to determine the estimated population proportion of MS in NF1 patients followed in our Referral Centre for Neurofibromatosis using the Informatics for Integrated Biology and the Bedside (i2b2) platform to extract information from the hospital’s electronic health records. We found a total 1507 patients with confirmed NF1, aged 18 years (y) and above (mean age 39.2y, range 18-88y; 57% women). Five NF1 individuals were found to have MS, yielding an estimated population proportion of 3.3 per 1000 (0.0033, 95% Confidence Interval 0.0014–0.0077). The median age at diagnosis was 45 y (range 28–49 y). Three patients had relapsing-remitting MS and two patients had secondary progressive MS. Patients with NF1 were found to be twice more likely to develop MS than the general population in France (odds ratio 2.2), however this result was not statistically significant (95% Confidence Interval 0.91–5.29). Our results show that patients with NF1 might have a slight increased tendency to develop MS; however, due to the small sample size of our study, the results may not be sufficiently powered to detect this rare association. Large-scale epidemiological studies based on nationwide datasets are needed to confirm our findings. These findings further emphasize the need for a focused follow-up of patients with NF1, as early detection and management of MS can prevent further neurological disability.

Dear Editor,

Neurofibromatosis 1 (NF1) is one of the most common autosomal dominant genetic disorders with a birth incidence as high as 1:2000 [1, 2]. It is caused by mutations in the *NF1* gene on chromosome 17 which encodes neurofibromin, a tumor suppressor protein [3, 4]. Neurofibromin regulates neuronal differentiation via its GTPase-activating protein function toward Ras [5]. By affecting multiple systems - including the cutaneous, neurologic, and orthopedic systems - NF1 leads to significant morbidity or mortality [2]. While NF1 individuals are predisposed to develop both benign and malignant nervous system tumors, various non-tumoral neurological conditions including learning disabilities [6] and attention deficit disorders [7] have also been reported to occur more frequently in NF1. Furthermore, NF1 has been linked to other chronic neurological conditions [8], such as epilepsy [9], sleep disorders [10], headaches [11], Parkinson’s disease [12], and multiple sclerosis [12].

Multiple sclerosis (MS) is the most common immune-mediated inflammatory demyelinating disease of the central nervous system (CNS) [13]. MS onset and course may depend on immunological, genetic and environmental factors [13]. Several case reports and small patient series have suggested an association between MS and NF1 [14–18]; however, these are limited by the lack of a non-NF1 comparison group. The number of epidemiologic studies on MS in patients with NF1 is very limited. Only one large case-control study using administrative claims data on 8579 NF1 individuals was performed and found a two-fold increased odds of MS-related health insurance claims in people with NF1 [12]. The aim of this study was to determine the estimated population proportion of multiple sclerosis in NF1 patients followed in our Referral Centre for Neurofibromatosis.

We used the Informatics for Integrated Biology and the Bedside (i2b2) platform to extract information from the hospital’s electronic health records (EHR). We performed a keyword search on clinical notes generated between Jan/01/2014 and Aug/02/2019 for patients aged 18 years (y) or older. Patients with NF1 were identified using the keyword “Neurofibromatosis”. The visit notes were then read thoroughly, and only patients with a confirmed NF1 diagnosis according to the National Institutes of Health criteria were included [19]. To identify NF1 patients with multiple sclerosis, the keywords “Multiple sclerosis” were entered using the Query Tool and an “and” relationship with Neurofibromatosis. Visit notes were then read to determine the NF1 phenotype, the age at diagnosis of MS, the MS subtype, the disease status at last follow-up visit, and the presence of another malignancy in the medical history. The NF1 phenotype was defined as a high-risk phenotype if the individual presented at least 10 subcutaneous neurofibromas and/or at least one internal neurofibroma [20, 21]. France is considered a country of medium-to-high MS prevalence and incidence. Prevalence of MS for the general population (1.5 per 1000) in France was obtained from an administrative data study from the main French health insurance system published in 2017 [22]. The study received institutional board review approval.

We found a total of 2215 patients with the term “Neurofibromatosis” written in their clinical notes. After reading each visit note thoroughly, a total of 1507 patients with confirmed NF1 aged 18y and above were identified (mean age 39.2y, range 18-88y; 57% women). Five NF1 individuals were found to have MS, yielding an estimated population proportion of 3.3 per 1000 (0.0033, 95% Confidence Interval 0.0014–0.0077). The median age at diagnosis was 45y ranging from 28 to 49y (average age 43y). Three patients had relapsing-remitting MS and two patients had secondary progressive MS. None of the patients had a high-risk phenotype. Patients with NF1 were found to be twice more likely to develop MS than the general population in France (odds ratio 2.2), however this result was not statistically significant (95% Confidence Interval 0.91–5.29).

Several case reports and case series have already indicated the potential association between NF1 and MS. [14–17] One large case-control study based on administrative claims data found a two-fold increased prevalence of MS in NF1 individuals; however, MS remains a rare complication of NF1 as it affects less than 1% of NF1 individuals (0.3% in our study).

A small body of evidence suggests that this association might have a molecular explanation. The NF1 gene is located on chromosome 17q, contains 60 exons and spans 350 kb of genomic DNA. This *NF1* gene is highly expressed in the myelin-forming oligodendrocytes which are the primary targets of the inflammatory and immune attacks in MS [23]. *NF1* gene inactivation in both mice [24] and zebrafish [25] results in increased oligodendrocyte precursor cells; and NF1 loss in in *NF1* genetically-engineered mouse models results in nitric oxide-mediated blood-brain barrier defects [26]. Furthermore, the gene for oligodendrocyte myelin glycoprotein *OMG* is embedded within intron 27b of the *NF1* gene. OMG is a membrane glycoprotein that appears in the human central nervous system at the time of myelination [27]. This raises the possibility that impaired OMG function could affect myelination, predisposing some NF1 individuals to MS. [18, 28] However, one study examined the *OMG* genes of four unrelated patients with NF1 and primary progressive MS and found that only two patients had alterations in the *OMG* gene. It was concluded that *OMG* mutations are neither necessary nor sufficient for primary progressive MS [18]. Moreover, one study found that a mutation in the *OMG* gene occurred in identical proportions in MS patients and healthy controls; making the *OMG* gene unlikely to be involved in the genetic susceptibility to MS [28].

Another suggested hypothesis relies in the role of the neurofibromin protein as a suppressor of cellular proliferation [29]. In NF1 patients, the absence of neurofibromin activity results in cell over-proliferation and tumor formation. Loss of NF1 in the Schwann cell lineage generates tumors composed of axonal processes, Schwann cells, fibroblasts, perineurial cells, and mast cells [30]. Hypothetically, if it also has a suppressor effect on the cells of the immune system, a loss of NF1 activity might result in over-activity of the immune system in some susceptible patients [31]. Furthermore, NF1 loss in *NF1* genetically-engineered mouse models was also found to cause an altered myelin structure [26]. So, it might be possible that in patients with NF1, exposure of the immune system to an altered myelin peripherally might activate an autoimmune response to the myelin expressed in the CNS. Further mechanistic analyses are needed to elucidate the relationship between NF1 and MS pathogenesis.

Our study further confirms that the range of MS associated with NF1 includes different forms of the disease. Indeed, earlier reports on the association of NF1 and MS described patients with primary progressive forms of MS [15, 18]; but afterwards, other forms such as relapsing–remitting and secondary progressive MS associated with NF1 have been reported [14, 16]. Accordingly, three of our patients had relapsing-remitting MS and one patient had secondary progressive MS.

An important strength of our study is that all patients had a definitive diagnosis of NF1. An important limitation is the small sample size, which led to results not sufficiently powered to detect such a rare association. Another limitation is that it is a retrospective study based on the EHR of our Referral Center for Neurofibromatosis; this hospital-based recruitment may have led to a selection bias, as patients with MS and/or more severe forms of NF1 are more prone to seek medical care in a referral center and, therefore, may lead to an overestimation of the prevalence of MS. However, our findings are consistent with previous reports.

To conclude, our results show that patients with NF1 might have an increased tendency to develop MS. These findings further emphasize the need for a focused follow-up of patients with NF1. Early detection of MS in NF1 individuals is crucial as early management could prevent further neurological disability. Large-scale epidemiological studies based on nationwide data sets are needed to confirm our findings.

## Data Availability

Data sharing not applicable to this article as no datasets were generated or analyzed during the current study.

## Abbreviations

NF1: Neurofibromatosis 1
MS: Multiple sclerosis
i2b2: Integrated Biology and the Bedside
CNS: Central nervous system
EHR: Electronic health records
